# Prediction of Overall Survival in Glioblastoma Using Early Postoperative Reduction in FLAIR Lesion Volume After Gross Total Resection

**DOI:** 10.3390/cancers18101585

**Published:** 2026-05-13

**Authors:** Takuma Aoki, Makoto Ohno, Go Horiguchi, Shunsuke Yanagisawa, Daisuke Kawauchi, Takaki Omura, Genta Fujii, Koji Saito, Naoya Hashimoto, Yoshitaka Narita

**Affiliations:** 1 Department of Neurosurgery and Neuro-Oncology, National Cancer Center Japan, Tokyo 104-0045, Japan; aokit@koto.kpu-m.ac.jp (T.A.); shuyanag@ncc.go.jp (S.Y.); dakawau2@ncc.go.jp (D.K.); tomura@ncc.go.jp (T.O.); gfujii917@gmail.com (G.F.); sks1224_65_zy@yahoo.co.jp (K.S.); 2 Department of Neurosurgery, Graduate School of Medical Science, Kyoto Prefectural University of Medicine, Kyoto 602-8566, Japan; nhashimo@koto.kpu-m.ac.jp; 3 Department of Biostatistics, Kyoto Prefectural University of Medicine, Kyoto 602-8566, Japan; go-h@koto.kpu-m.ac.jp

**Keywords:** glioblastoma, FLAIR lesion volume, residual tumor, overall survival, tumor volume reduction

## Abstract

**Background/Objectives**: Glioblastoma (GBM) comprises a contrast-enhancing (CE) mass and peritumoral hyperintensity on fluid-attenuated inversion recovery (FLAIR) on magnetic resonance imaging (MRI). Although a smaller residual FLAIR lesion volume (FLV) after gross total resection (GTR) is associated with longer survival, the clinical observation of rapid FLV reduction within the first postoperative month remains unknown. We evaluated the independent prognostic contributions of early and late FLV reduction in patients with newly diagnosed GBM, isocitrate dehydrogenase (IDH)-wildtype, who underwent GTR. **Methods**: In a retrospective cohort of 51 adults with GBM, IDH-wildtype, who underwent GTR and standard chemoradiotherapy, semi-automated FLV was measured on MRI preoperatively, immediately postoperatively (FLV0), at 1 month (FLV1), and at 3 months (FLV3). FLV changes were expressed as percentage changes relative to the preceding timepoint (ΔFLV0-1, ΔFLV1-3). Using a 3-month landmark analysis, multivariable Cox models for overall survival (OS) were fitted, adjusted for age, *O6-methylguanine-DNA-methyltransferase* promoter (p*MGMT*) methylation, and postoperative Karnofsky Performance Status (KPS). **Results**: In the multivariable 3-month landmark analysis, each 10-percentage-point early FLV reduction (∆FLV0-1^[10-pp]^) was associated with lower mortality risk (hazard ratio [HR] 0.91, 95% confidence interval [CI] 0.82–0.99, *p* = 0.037) whereas a late FLV reduction (∆FLV1-3^[10-pp]^) showed a smaller association with OS (HR 0.99, 95% CI 0.98–1.00, *p* = 0.043). In an exploratory dichotomized analysis at a 20% reduction threshold, early responders (∆FLV0-1 ≥ 20%) had markedly better OS (HR 0.33, *p* = 0.010). **Conclusions**: In selected patients with GBM, IDH-wildtype, who achieved radiographic GTR and received standard chemoradiotherapy, early postoperative FLV reduction at 1 month independently predicted longer OS, with a substantially larger effect than late reduction. Prospective multi-center validation is required before clinical implementation.

## Simple Summary

Glioblastoma is an aggressive primary brain tumor. After surgical resection, the peritumoral fluid-attenuated inversion recovery (FLAIR) hyperintense region on magnetic resonance imaging reflects a mixture of residual infiltrating tumor, vasogenic edema, and postoperative inflammatory changes—components that cannot be reliably distinguished on standard imaging. Although a smaller residual FLAIR volume is associated with better prognosis, whether the timing of FLAIR volume reduction carries distinct prognostic information remains unclear. In 51 patients with glioblastoma, isocitrate dehydrogenase wildtype, who underwent gross total resection, early FLAIR volume reduction within the first postoperative month was independently associated with improved overall survival, whereas late reduction between 1 and 3 months showed a substantially smaller association. This temporal difference suggests that early FLAIR dynamics may reflect the biological composition of the residual region rather than treatment responsiveness. In patients with limited early reduction, closer surveillance and earlier consideration of treatment intensification may be warranted. These findings require validation in larger prospective studies.

## 1. Introduction

Glioblastoma (GBM) is the most common primary malignant brain tumor of the central nervous system (CNS) and accounts for 15–20% of all CNS and 50–60% of malignant CNS tumors [1]. Standard therapy comprises maximal safe resection of the contrast-enhancing (CE) lesion followed by radiotherapy (RT; 60 Gy or hypofractionated 40 Gy) with concomitant and adjuvant temozolomide, with or without tumor-treating fields (TTFields) [2,3,4]. Despite multimodal treatment, the prognosis remains poor, with a median overall survival (OS) of approximately 15 months (range 14–21) and a median progression-free survival (PFS) of approximately 10 months [2,5,6].

The established prognostic factors include patient age, preoperative and postoperative Karnofsky Performance Status (KPS), extent of resection, and *O*^6^*-methylguanine-DNA-methyltransferase* promoter (p*MGMT*) methylation status [7,8,9,10,11,12,13,14,15]. On magnetic resonance imaging (MRI), GBM typically shows both CE tumors on T1-weighted images and peritumoral hyperintensity on T2/fluid-attenuated inversion recovery (FLAIR) images. This peritumoral FLAIR signal likely represents a heterogeneous mixture of vasogenic edema, postoperative inflammatory and ischemic changes, and infiltrating non-contrast-enhancing (nCE) tumor cells—components that cannot be reliably distinguished on standard MRI alone [16,17,18].

The prognostic significance of the residual FLAIR/nCE burden after resection has been established by the Response Assessment in Neuro-Oncology (RANO) resect group. Their volumetric classification system distinguishes Class 1 (complete CE resection with ≤5 cm^3^ nCE residuum) from Class 2A (complete CE resection with >5 cm^3^ nCE residuum), and a smaller residual nCE volume—with ≤40 mL as a clinically relevant threshold—has been associated with longer survival [17,19]. Furthermore, recurrent GBM spreads predominantly along white matter tracts, and the spatial relationship between the FLAIR region and fiber tract architecture may influence both residual tumor burden and recurrence patterns [20].

Despite these advances, a critical question remains unanswered. Does the timing of postoperative FLV reduction carry distinct prognostic information? Most prior studies have assessed residual FLV at a single timepoint, typically 2–6 months after chemoradiotherapy (CRT), and RANO 2.0 recommends the 3-month post-surgical MRI as the new baseline for response evaluation [21,22,23,24]. However, in clinical practice, rapid FLV reduction is often observed during the early postoperative period before the 3-month baseline, and the prognostic significance of this early FLV change remains poorly understood.

In this study, we aimed to investigate the prognostic significance of early (0–1 month, ∆FLV0-1) versus later (1–3 months, ∆FLV1-3) postoperative FLV reduction in patients with GBM, isocitrate dehydrogenase (IDH)-wildtype, who achieved radiographic gross total resection (GTR). The central question was whether the two temporal windows carry distinct prognostic weight and whether their difference can inform the biological composition of the residual FLAIR region. We hypothesized that early FLV reduction—occurring prior to meaningful CRT effect—primarily reflects vasogenic edema resolution, and that a greater early reduction therefore indicates a lower residual infiltrative tumor fraction within the FLAIR region.

## 2. Materials and Methods

### 2.1. Study Design and Patient Selection

We conducted a retrospective cohort study of consecutive adults with newly diagnosed GBM, IDH-wildtype, who were treated at our institution between 2010 and 2024. To minimize the survival advantage attributable to the surgical extent of resection, we restricted the cohort to patients who achieved radiological gross total resection (GTR) on postoperative MRI [25]. Mutations in *IDH 1/2*, *Telomerase Reverse Transcriptase* (*TERT)* promoter (*C228T/C250T*), and *B-Raf proto-oncogene* (*BRAF) V600E* were analyzed by Sanger sequencing or next-generation sequencing, and the p*MGMT* methylation status was analyzed by pyrosequencing. Hyper p*MGMT* methylation was defined as >10% [6,26]. All patients received concurrent chemoradiotherapy with temozolomide. Radiotherapy was delivered via three-dimensional conformal radiation therapy (3D-CRT) or intensity-modulated radiation therapy (IMRT). The prescribed total dose ranged from 40 to 60 Gy and was administered using standard fractionation or hypofractionated regimens, mainly for older patients ≥ 70 years. The radiation fields were planned as involved-field irradiation based on postoperative imaging and institutional protocols.

Inclusion criteria

1. Age ≥ 18 years.
2. Postoperative KPS ≥ 70.
3. A solitary contrast-enhancing lesion on preoperative MRI.
4. Histopathologic confirmation of GBM, IDH-wildtype, according to the WHO 2021 criteria.
5. Radiographic GTR of all CE lesions confirmed on the postoperative scan.
6. Availability of postoperative serial MRI with interpretable image quality preoperatively, postoperatively, and at approximately 1 and 3 months.
7. Except for the first week after surgery, steroids were not administered during the 3-month follow-up period, unless required for non-oncological indications.

Exclusion criteria

We excluded predominantly cystic CE lesions, cases with radiologic dissemination on preoperative MRI, and cases treated with bevacizumab within 3 months postoperatively.

### 2.2. MRI Acquisition and Volumetry

T1-weighted contrast-enhanced (CE) and T2/FLAIR MRI were obtained using 1.5- or 3.0-Tesla scanners at four timepoints: preoperative (preOp), immediately postoperative, 1 month, and 3 months. We defined two volumetric measures: contrast-enhancing volume (CEV), the volume of the enhancing tumor on CE, and FLAIR hyperintense volume (FLV), the volume of the non-enhancing peritumoral hyperintensity on FLAIR. The CEV was measured using preOp T1CE. FLV was measured preoperatively (PreOp), immediately postoperatively (within 48 h of surgery; FLV0), at 1 month (FLV1), and at 3 months (FLV3) (Figure S1, top panel). With the outcomes blinded, a board-certified neurosurgeon (TA) performed semi-automated 3D volumetry using a dedicated platform (Synapse Vincent^®^ version6, Fujifilm Corp., Tokyo, Japan), with manual review/correction as needed (Figure S1, bottom panel). The use of semi-automated segmentation minimized subjective variability in volume measurements.

The percentage changes in FLV were computed as:

Early 0–1% change:

$$\Delta \mathrm{FLV}0\text{-}1(\%)\;=\;100\;\times \;\frac{FLV0\;-\;FLV1\;}{FLV0}$$

Delayed 1–3% change:

$$\Delta \mathrm{FLV}1\text{-}3(\%)\;=\;100\;\times \;\frac{FLV1\;-\;FLV3\;}{FLV1}$$

Cumulative 0–3% change:

$$\Delta \mathrm{FLV}0\text{-}3(\%)\;=\;100\;\times \;\frac{FLV0\;-\;FLV3\;}{FLV0}$$

Positive values indicated a reduction (shrinkage) in the FLV over the specified interval, whereas a larger positive percentage denoted a greater absolute decrease.

For survival modeling, FLV reduction was treated as a continuous predictor and scaled so that the hazard reflects a 10-percentage-point (10-pp) greater FLV reduction, as follows.

$$∆FLV0\text{-}1^{\left[10pp\right]}=∆FLV0\text{-}1(\%)/10,\;∆FLV1\text{-}3^{\left[10pp\right]}=∆FLV1\text{-}3(\%)/10,\;∆FLV0\text{-}3^{\left[10pp\right]}=∆FLV0\text{-}3(\%)/10$$

### 2.3. Study Endpoints

The primary endpoint was the OS from surgery to death, and survivors were censored at the last follow-up. The secondary endpoint was PFS, which based on the RANO 2.0 criteria, was dated as the first qualifying event that was adjudicated jointly by neuroradiologists and neurosurgeons [27].

### 2.4. Statistical Analysis

#### Primary Survival Analysis

A 3-month landmark analysis was conducted to minimize immortal-time bias. Only patients alive at 3 months were included, with risk time starting from this landmark. Exposures were percentage FLV reductions during the early window (∆FLV0-1) and delayed window (∆FLV1-3), each expressed per 10 pp decrease. The functional form of the log hazard ratio (HR) was evaluated using a restricted cubic spline (RCS) model. No evidence of nonlinearity was observed within the 10th–90th percentile range; therefore, FLV reduction was modeled as a continuous variable per 10-percentage-point (10-pp) decrease. Multivariable Cox proportional hazards models were prespecified and adjusted for clinically relevant covariates, including age (≤65 years), p*MGMT* methylation status, and postoperative KPS ≥ 80 [9,10,14,19,28]. Cluster-robust standard errors were used, with the patient ID as clusters. The proportional hazard assumption was assessed using Schoenfeld residuals. The correlation between ΔFLV0-1 and ΔFLV1-3 was assessed using Pearson’s correlation coefficient, and variance inflation factors (VIFs) were calculated using a linear regression model to evaluate multicollinearity. To formally compare the prognostic contributions of the two windows, both ΔFLV0-1 and ΔFLV1-3 were entered simultaneously into the multivariable model, and the difference between their coefficients was tested using a linear combination test. A sensitivity analysis was performed by adding preoperative FLV as an additional covariate to the primary model.

For clinical interpretability, an exploratory dichotomized analysis was performed using a 20% reduction threshold as a clinically intuitive approximation of a meaningful volumetric response, whereas the continuous specification remained the primary specification. To assess the robustness of the 20% threshold, sensitivity analyses were performed using alternative thresholds of 10%, 15%, 25%, and 30%.

All tests were two-sided with α = 0.05, and *p* < 0.05 was considered statistically significant.

### 2.5. Additional Survival Analyses

In RANO Class 2A patients, Cox proportional hazards models treated ΔFLV0-1 and ΔFLV1-3 as continuous predictors, with linearity confirmed by restricted cubic splines. Multivariable models were adjusted for p*MGMT* status, postoperative KPS, age, and residual FLV0 dichotomized at 40 mL—a threshold previously reported to be associated with survival in the RANO resect classification [17]. Patients were further stratified by FLV0 (≤40 vs. >40 mL) for exploratory Kaplan–Meier analyses.

All analyses were performed in Stata 18 (StataCorp, College Station, TX, USA).

## 3. Results

### 3.1. Patients and Volumetric MRI Measures

We screened 60 consecutive adults with newly diagnosed glioblastoma who underwent GTR for all CE lesions, and nine patients were excluded according to the prespecified criteria as follows: predominantly cystic CE lesion (*n* = 1), radiological dissemination (*n* = 2), and use of bevacizumab within 3 months after surgery (*n* = 6). The patient enrollment and exclusion process is summarized in Figure S2. The remaining 51 patients were included in the final analysis (Table 1). Two patients received corticosteroids during the 3-month postoperative period (excluding routine perioperative use within the first postoperative week). In both cases, steroid use was temporary and did not reflect radiographic tumor progression; one patient received steroids for skin rash, and the other required a tapering course extending beyond the first postoperative week. Only one patient experienced recurrence within 3 months after surgery.

The median age was 63.0 years; 29 patients (56.9%) were male, 49 patients (96.1%) had preoperative KPS ≥ 70, and 44 patients (86.3%) had postoperative KPS ≥ 80. The median OS and PFS after the first surgery were 23.0 and 10.4 months, respectively. Hyper p*MGMT* methylation status was observed in 33.3% of all patients. The immediate postoperative MRI was obtained at a median of 1 day (Interquartile Range; IQR 1–2) after surgery, and the 1-month and 3-month MRIs were obtained at a median of 35 days (IQR 27–39) and 102 days (IQR 84–134), respectively.

The pattern of first recurrence was local in 37 patients (72.5%), distant in 7 patients (13.7%), leptomeningeal dissemination in 4 patients (7.8%), and no recurrence in 3 patients (5.9%). Among the patients who developed local recurrence, the median time to local recurrence (local PFS) was 7.9 months.

According to the RANO categories, 3 patients (5.9%) were classified as Class 1 and 48 patients (94.1%) as Class 2A. RANO risk scores were low in 16 patients (31.4%) and intermediate in 35 patients (68.6%). The baseline volumetric MRI measurements are summarized in Table S1 and Figure S3A. The postoperative FLV tended to decrease during the early interval (FLV0 to FLV1) in most patients, whereas the late interval (FLV1 to FLV3) showed mixed changes with frequent re-expansion. Consequently, the net change over the first 3 months (FLV0 to FLV3) was largely driven by early postoperative changes.

### 3.2. Primary Analysis

For the 3-month landmark analysis, one patient was censored before 3 months and excluded, leaving 50 patients. RCS evaluation confirmed approximate linearity of OS within the 10th–90th percentile range for both ΔFLV0-1 and ΔFLV1-3; thus, FLV reduction was modeled as a continuous variable per 10 pp decrease. Univariate results are in Table S2. In the multivariable landmark model, a 10 pp greater ΔFLV0-1 was associated with a lower mortality risk (HR 0.91, 95% CI 0.82–0.99, *p* = 0.037; Model A in Table 2), whereas ΔFLV1-3 showed a statistically significant but substantially smaller association with OS (HR 0.99, 95% CI 0.98–1.00, *p* = 0.043; Model B). Age ≤ 65 years (HR 0.32) and p*MGMT* methylation (HR 0.30) were independently associated with better prognosis.

For PFS, RCS analyses suggested potential nonlinearity in ΔFLV0-1; FLV change metrics were not modeled as linear continuous predictors for PFS.

### 3.3. Early Change vs. Delayed Change

**Figure 1:**
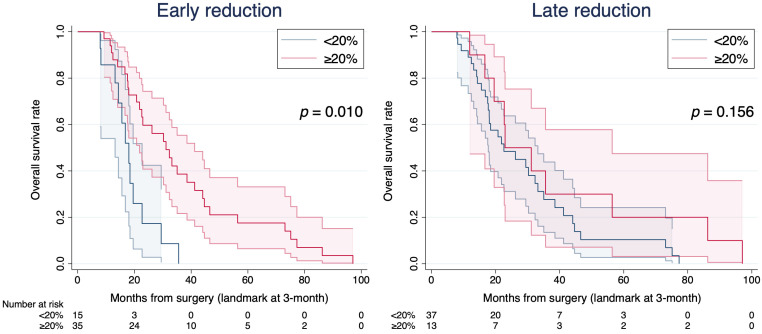
Exploratory Kaplan–Meier analysis of overall survival by early and late FLV reduction (20% cutoff for clinical interpretability). Exploratory Kaplan–Meier curves for overall survival at the 3-month (3M) landmark according to early (ΔFLV0-1) and late (ΔFLV1-3) FLV reduction (*n* = 50), dichotomized at a 20% threshold chosen for clinical interpretability. This cutoff should not be interpreted as a threshold for risk classification. Shaded areas represent 95% confidence intervals.

To directly compare the prognostic contributions of the two windows, ΔFLV0-1 and ΔFLV1-3 were entered simultaneously into the multivariable landmark model. Each 10 pp greater reduction in FLV was associated with an HR of 0.90 in the early window (ΔFLV0-1) and 0.99 in the delayed window (ΔFLV1-3). This indicates a substantially larger prognostic effect for early reduction. For interpretability, dichotomizing each window at ≥20% showed that early ≥20% was associated with better OS in the landmark Kaplan–Meier analysis (log-rank *p* = 0.001) and in the adjusted landmark model (HR 0.33, *p* = 0.010), whereas the delayed reduction of ≥20% was not significant (log-rank *p* = 0.156, adjusted HR 0.48, *p* = 0.093, Figure 1, Table S3).

The correlation between ΔFLV0-1 and ΔFLV1-3 was low (r = 0.059), and all VIFs were all below 1.2 (mean VIF 1.12), indicating no evidence of multicollinearity. When both variables were entered simultaneously, the difference between their coefficients was statistically significant (*p* = 0.020), confirming that early reduction had a substantially larger prognostic effect than delayed reduction. A sensitivity analysis adding preoperative FLV as a covariate did not materially change the results (HR 0.901, 95% CI 0.828–0.982, *p* = 0.017). Sensitivity analyses using alternative dichotomization thresholds (10%, 15%, 25%, and 30%) consistently showed that early reduction was associated with improved OS across all thresholds, whereas late reduction did not reach statistical significance at any threshold.

### 3.4. FLV Dynamics in RANO Class 2A

**Figure 2:**
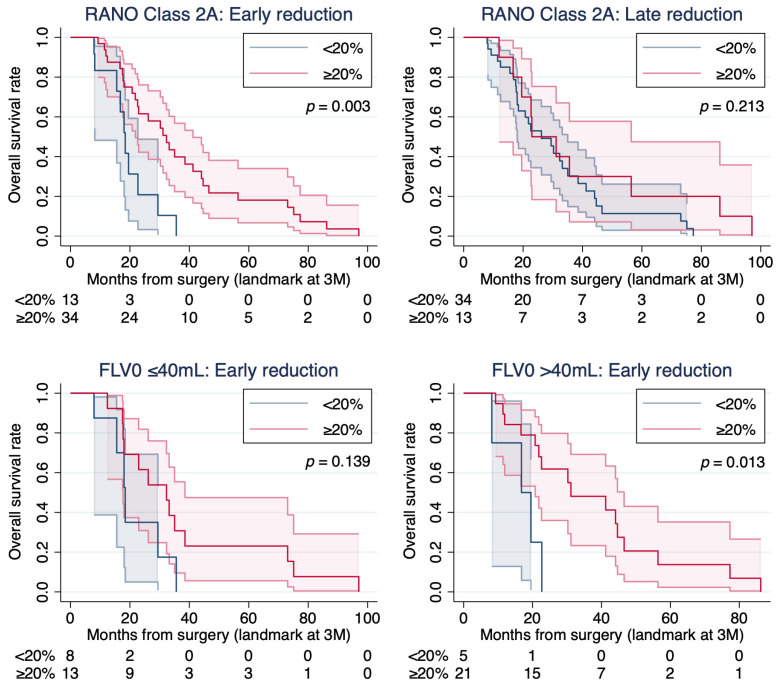
Exploratory Kaplan–Meier analysis of overall survival by FLV reduction in Response Assessment in Neuro-Oncology (RANO) Class 2A patients (20% cutoff for clinical interpretability). Exploratory Kaplan–Meier curves for overall survival according to FLV reduction in RANO Class 2A patients, dichotomized at a 20% threshold chosen for clinical interpretability. (Top left) Early reduction, all Class 2A (*n* = 47; log-rank *p* = 0.003). (Top right) Late reduction, all Class 2A (*n* = 47; log-rank *p* = 0.213). (Bottom left) Early reduction, FLV0 ≤ 40 mL (*n* = 21; log-rank *p* = 0.139). (Bottom right) Early reduction, FLV0 > 40 mL (*n* = 26; log-rank *p* = 0.013). Shaded areas represent 95% confidence intervals. All results should be interpreted with caution given the exploratory nature of the analysis and the limited sample size.

In RANO Class 2A patients at the 3-month landmark (*n* = 47), RCS evaluation confirmed approximate linearity for both ΔFLV0-1 and ΔFLV1-3; thus, both were modeled as continuous variables. Early FLV reduction was independently associated with improved OS in univariate (HR 0.902, *p* = 0.028) and multivariable analyses adjusted for FLV0 (≤40 vs. >40 mL), p*MGMT* status, age, and postoperative KPS (HR 0.90, *p* = 0.037; Table S4). The association for delayed reduction did not reach statistical significance (HR 0.993, *p* = 0.060). Exploratory Kaplan–Meier analyses dichotomized at ≥20% showed consistent findings in the overall Class 2A cohort (Figure 2, top panel). In both FLV0 subgroups (≤40 and >40 mL), early reduction tended to be associated with more favorable OS. The association reached statistical significance in the FLV0 > 40 mL stratum (log-rank *p* = 0.013), whereas it remained a trend in the FLV0 ≤ 40 mL stratum (log-rank *p* = 0.139; Figure 2, bottom panels).

## 4. Discussion

The central finding of this study is that early postoperative FLV reduction (ΔFLV0-1, 0–1 month) carries substantially greater prognostic weight than late reduction (ΔFLV1-3, 1–3 months) in patients with IDH-wildtype GBM who achieved radiographic GTR. Each 10 pp greater early reduction was associated with a 10% reduction in mortality risk, and the difference in prognostic effect between the two temporal windows was statistically significant (*p* = 0.020), approximately a ten-fold difference in effect size. This temporal distinction, rather than the FLV-survival association per se, represents the novel contribution of this work. Consistent findings were observed in the RANO Class 2A subgroup, including patients with large residual FLV, in which early reduction remained prognostically significant while the contribution of late reduction was limited. These results suggest that the timing of FLV assessment matters. The 1-month postoperative MRI may capture prognostically relevant tumor biology that is not reflected in the standard 3-month baseline, in this specific population of GTR-treated GBM, IDH-wildtype patients.

### 4.1. FLV Reduction

Prior studies of GBM demonstrated that larger postoperative FLV assessed at 2–6 months after CRT was associated with worse OS and PFS [21,22], and that smaller residual FLV at 3 months (<19.3 cm^3^ or <46% of baseline) correlated with longer survival [23]. However, these studies evaluated heterogeneous surgical cohorts at later timepoints and did not examine whether the FLV trajectory in the early postoperative window—before the influence of CRT—carries independent prognostic value. By restricting our cohort to patients who achieved radiographic GTR and separating net FLV change into early (ΔFLV0-1) and late (ΔFLV1-3) components, we were able to demonstrate that the early postoperative FLV trajectory is independently associated with OS. In our cohort, the FLV decreased in most patients during the early window (41/51) while the late window showed mixed changes (22/51 decreases, 29/51 increases). Among patients with net 0–3-month decreases, the early component accounted for the majority of the overall reduction (Figure S3B). Clinically, early postoperative FLV reduction can help determine whether closer surveillance or intensified treatment, such as reoperation or the addition of bevacizumab, is indicated.

### 4.2. Biological Interpretation

The biological basis of early FLV reduction remains incompletely understood, and its interpretation requires caution. The peritumoral FLAIR signal possibly comprises both vasogenic edema and inflammatory changes, as well as infiltrative tumor cells, components that cannot be reliably distinguished on standard MRI alone. The RANO resect group demonstrated that residual FLV is associated with prognosis, with raters attempting to distinguish edema-like from tumor-like FLAIR patterns using imaging features [17,19]. However, it remains uncertain what proportion of postoperative FLAIR abnormality truly represents infiltrative nCE tumor versus reversible perioperative tissue changes. We hypothesize that a greater early decrease in FLV (ΔFLV0-1) primarily reflects the rapid resolution of vasogenic edema and perioperative inflammatory changes, which would, in turn, indirectly indicate a lower residual infiltrative tumor fraction within the FLAIR region. This hypothesis is supported by the temporal dissociation from CRT effects, but cannot be confirmed without histological or advanced imaging validation. Notably, systemic inflammatory state may also influence peritumoral MRI signal dynamics during the early postoperative period, representing an additional biological variable that future studies should address [29].

### 4.3. Early Reduction vs. Late Reduction

**Figure 3:**
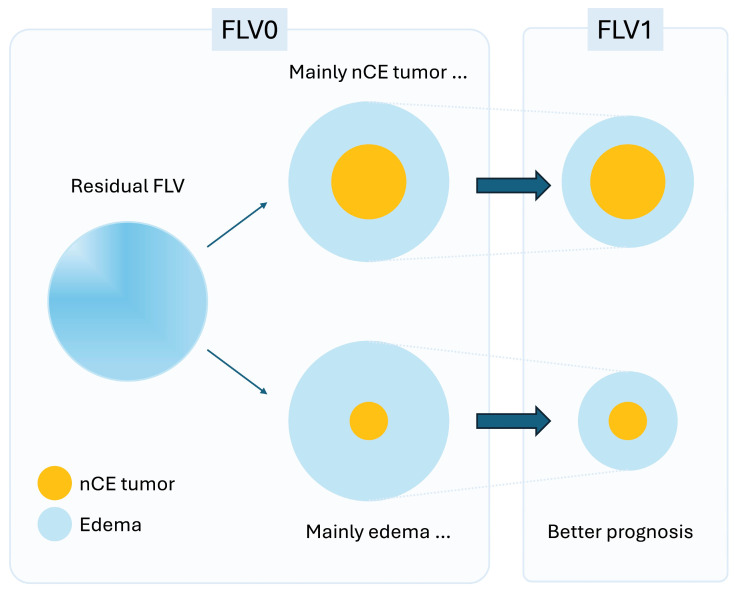
Conceptual schematic linking early FLV reduction to prognosis. Residual postoperative FLAIR hyperintensity (FLV0) possibly comprises a mixture of resolving components (e.g., vasogenic edema and inflammation) and less-resolving components (putative nCE tumors). When the residual FLV is edema-dominant, FLV decreases substantially from FLV0 to FLV1 (larger ΔFLV0-1 reduction), which is associated with longer overall survival in our cohort. However, when the residual FLV contained a higher fraction of less-resolving components, early reduction was limited (smaller ΔFLV0-1 reduction), potentially indicating a higher residual infiltrative tumor burden. This figure presents a conceptual schematic proposed by the authors to illustrate a working hypothesis and does not represent direct data from the present study.

The significance of postoperative FLV dynamics likely differs based on the timing of assessment. The early window (ΔFLV0-1) largely precedes the full biological effect of CRT, although it is important to acknowledge that chemoradiotherapy was initiated at a median of 17 days after surgery—within the early interval—and therefore treatment-related changes cannot be entirely excluded. Nevertheless, we interpret the dominant driver of early FLV reduction as vasogenic edema resolution rather than treatment response: CRT-mediated tumor response requires weeks of treatment, and any meaningful anti-tumor effect within the first month would be expected to be minimal. This interpretation is consistent with reports that surgical resection facilitates rapid edema resolution in brain tumors [30,31]. Rapid early FLV reduction may therefore reflect a favorable initial state in which the residual infiltrative tumor burden within the FLAIR region is inherently low; conversely, failure of early FLV to decrease may indicate a larger residual infiltrative tumor fraction. The late change (ΔFLV1-3), by contrast, temporally coincides with the completion of CRT and is more plausibly interpreted as a therapeutic response. That the early component was a substantially more potent prognostic driver than the late component suggests that prognosis in GTR-treated GBM may be predominantly determined by the pre-treatment residual infiltrative tumor burden, rather than by subsequent therapeutic responsiveness (Figure 3).

Nonetheless, patients with ΔFLV0-1 ≥20% included a higher proportion of p*MGMT* methylation (Table S5; Fisher’s exact *p* = 0.168), and we cannot fully exclude the possibility that early FLV reduction partly reflects treatment sensitivity to initial CRT. However, because this reduction occurs within weeks of surgery—before a meaningful CRT effect would be expected—we interpret it as more consistent with vasogenic edema resolution, while acknowledging residual confounding by p*MGMT* status.

### 4.4. Limitations

This study has several limitations that should be considered when interpreting the findings. First, the retrospective, single-center design with a small, highly selected cohort—restricted to GTR-achieved, GBM, IDH-wildtype patients—limits generalizability. All findings, particularly those from subgroup analyses, are hypothesis-generating and require validation in larger, independent, multi-center cohorts before clinical conclusions can be drawn. Second, the postoperative FLAIR signal represents a heterogeneous mixture of vasogenic edema, inflammatory and ischemic changes, and infiltrating tumor cells that cannot be reliably separated on standard MRI. The biological interpretation of FLV dynamics therefore remains inferential. Third, residual confounding from unmeasured variables, including tumor location, degree of peritumoral infiltration, molecular heterogeneity beyond MGMT status, and heterogeneity in postoperative management, cannot be excluded. Fourth, volumetric measurements were performed by a single reader with outcomes blinded. The semi-automated methodology and outcome-blinded design partially mitigate observer-related variability, but independent multi-reader validation should be incorporated in future studies. Fifth, variability in scan timing across the early and late intervals and potential heterogeneity in scanner field strength across serial examinations may have introduced measurement variability, although most examinations were performed at our institution under standardized protocols, which partially mitigates this concern. Sixth, chemoradiotherapy was initiated at a median of 17 days after surgery, overlapping with the early interval. Therefore, treatment-related FLV changes cannot be entirely excluded, although we consider edema resolution the more likely dominant mechanism. Finally, the cohort spans 14 years (2010–2024), during which advances in surgical technique, radiation planning, MRI acquisition protocols, and supportive care may have introduced temporal heterogeneity that could not be fully accounted for in the analysis.

## 5. Conclusions

In a selected cohort of patients with GBM, IDH-wildtype, who achieved radiographic GTR and received standard CRT, early postoperative FLV reduction at 1 month independently predicted longer OS, with a substantially larger effect than late reduction. Given that the postoperative FLAIR region represents a heterogeneous mixture of vasogenic edema and infiltrative tumor, this temporal difference suggests that early FLV dynamics may reflect the relative contributions of these components rather than treatment responsiveness. In patients with limited early FLV reduction, closer surveillance and earlier consideration of treatment intensification may be warranted. These findings are hypothesis-generating and require prospective multi-center validation before clinical implementation.

## Figures and Tables

**Table 1:** Patient characteristics, postoperative treatment, and molecular profile.

Patient Characteristics	Total *n* = 51 (%)
Gender.	
Male	29 (56.9)
Female	22 (43.1)
Age	63.0
≤65	29 (56.9)
>65	22 (43.1)
Preoperative KPS	
90–100	27 (52.9)
80	17 (33.3)
70	5 (9.8)
60	2 (3.9)
Postoperative KPS	
90–100	26 (51.0)
80	18 (35.3)
70	7 (13.7)
OS, median, months	23.0
PFS, median, months	10.4
Postoperative therapy	
TMZ+RT(60 Gy)	45 (88.2)
TMZ+RT(45 Gy)/(40 Gy)	2/4 (11.8)
TTF	
use	2 (3.9)
no use	49 (96.1)
BEV use	
yes	34 (66.7)
no	17 (33.3)
start within 3 months	0 (0)
Start date of postoperative TMZ+RT, median, days	17
Molecular Characteristics	
MGMT	
hyper methylation	17 (33.3)
hypo methylation	34 (66.7)
IDH-WT	51 (100)
TERT	
WT	16 (31.4)
C228T/C250T	23/9 (62.7)
ND	3 (5.9)
BRAF	
WT	47 (92.2)
V600E	1 (2.0)
ND	3 (5.9)
RANO categories for EOR	
Class 1	3 (5.9)
Class 2A	48 (94.1)
RANO risk score	
low	16 (31.4)
intermediate	35 (68.6)
MRI timing, median (days, IQR)	
Preoperation	4 (2–9)
FLV0	1 (1–2)
FLV1	35 (27–39)
FLV3	102 (84–134)

* Baseline clinical, treatment, and molecular characteristics of the cohort (*n* = 51). Values are presented as *n* (%) unless otherwise indicated; continuous variables are shown as medians. In the RANO classification for extent of resection, Class 1 indicates supramaximal resection of the contrast-enhancing (CE) tumor (0 cm^3^ CE + ≤5 cm^3^ non-CE), whereas Class 2A indicates complete CE resection (0 cm^3^ CE + >5 cm^3^ non-CE). The RANO risk score was calculated based on the RANO resection class, postoperative KPS, age at resection, and p*MGMT* methylation status.

**Table 2:** Multivariable Cox proportional hazards models for overall survival at the 3-month landmark (separate models for early and late FLV reduction).

Model	Variable	HR	95% CI	*p*-Value
Model A	ΔFLV0-1^[10-pp]^	0.91	0.82–0.99	0.037
Model B	ΔFLV1-3^[10-pp]^	0.99	0.98–1.00	0.043

HRs are expressed per 10 pp absolute reduction in FLV. Two separate multivariable Cox models were fitted: Model A included ΔFLV0-1/10-pp and Model B included ΔFLV1-3/10-pp. Both models were specified a priori and adjusted for age ≤ 65 years, p*MGMT* methylation, and postoperative KPS ≥ 80. Cluster-robust standard errors were applied at the patient level. In Model A, greater early FLV reduction (0–1 months) was associated with improved overall survival. In Model B, late FLV reduction (1–3 months) showed a statistically significant but substantially smaller association with overall survival.

## Data Availability

De-identified data supporting the findings of this study are available from the corresponding author upon reasonable request. Access is subject to approval by the Institutional Review Board of the National Cancer Center Japan (protocol code 2013-042) and execution of a data sharing agreement, in accordance with applicable Japanese privacy regulations.

## Supplementary Materials

The following supporting information can be downloaded at: https://www.mdpi.com/article/10.3390/cancers18101585/s1, Figure S1: Imaging timeline, volumetry workflow, and definitions of FLV change metrics; Figure S2: Patient enrollment and exclusion flowchart. Figure S3. Distribution and direction of postoperative FLV changes; Table S1: Baseline volumetric MRI measures; Table S2: Univariable Cox regression at the 3-month landmark (overall survival); Table S3: Overall survival at the 3-month landmark: dichotomized FLV reduction (≥20% vs. <20%); Table S4: Multivariable Cox model at the 3-month landmark (OS) in RANO Class2 (47 patients with 39 death events/39 deaths); Table S5: Association between pMGMT methylation status andΔFLV01 in RANO Class2.

## Abbreviations

The following abbreviations are used in this manuscript:

BEV	Bevacizumab
CE	Contrast-enhancing
CEV	Contrast-enhancing volume
CI	Confidence interval
CRT	Chemoradiotherapy
CNS	Central nervous system
FLAIR	Fluid-attenuated inversion recovery
FLV	FLAIR lesion volume
GBM	Glioblastoma
GTR	Gross total resection
HR	Hazard ratio
IDH	Isocitrate dehydrogenase
KPS	Karnofsky performance status
MGMT	O6-methylguanine-DNA methyltransferase
MRI	Magnetic resonance imaging
nCE	Non-contrast-enhancing
OS	Overall survival
PFS	Progression-free survival
pp	Percentage points
RANO	Response Assessment in Neuro-Oncology
RCS	Restricted cubic spline
RT	Radiation therapy
TMZ	Temozolomide
TTFields	Tumor-treating fields
VIF	Variance inflation factor
