# Supplementary material for: Prediction of Overall Survival in Glioblastoma Using Early Postoperative Reduction in FLAIR Lesion Volume After Gross Total Resection
Source: Cancers (Basel). 2026 May 13;18(10):1585. doi: 10.3390/cancers18101585 (PMC13204664; doi:10.3390/cancers18101585)
Supplement: Supplementary file 1 [file cancers-18-01585-s001.zip › cancers-4272157-supplementary.pdf]

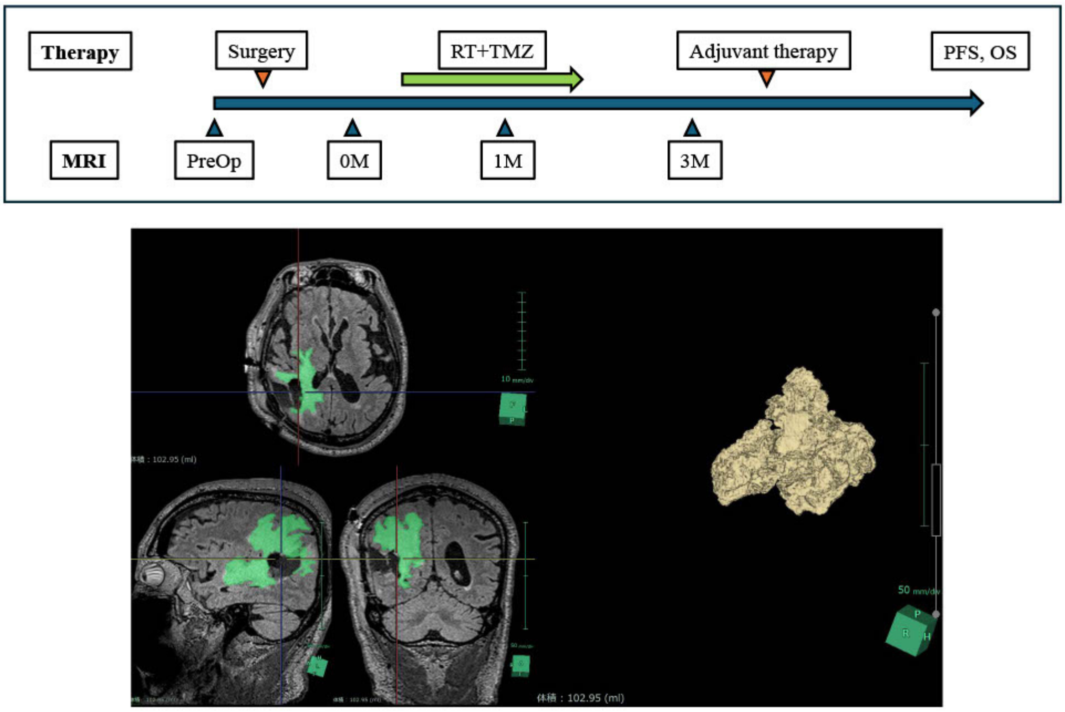

Figure S1. Imaging timeline, volumetry workflow, and definitions of FLV change metrics.

(Top) Study imaging timeline showing preoperation (PreOp), immediately postoperatively (0M), approximately 1 month (1M), and approximately 3 months (3M). (Bottom) Representative semi-automated 3D volumetry on T1CE and FLAIR (Synapse Vincent®, Fujifilm) with manual and semi-automatic quality control.

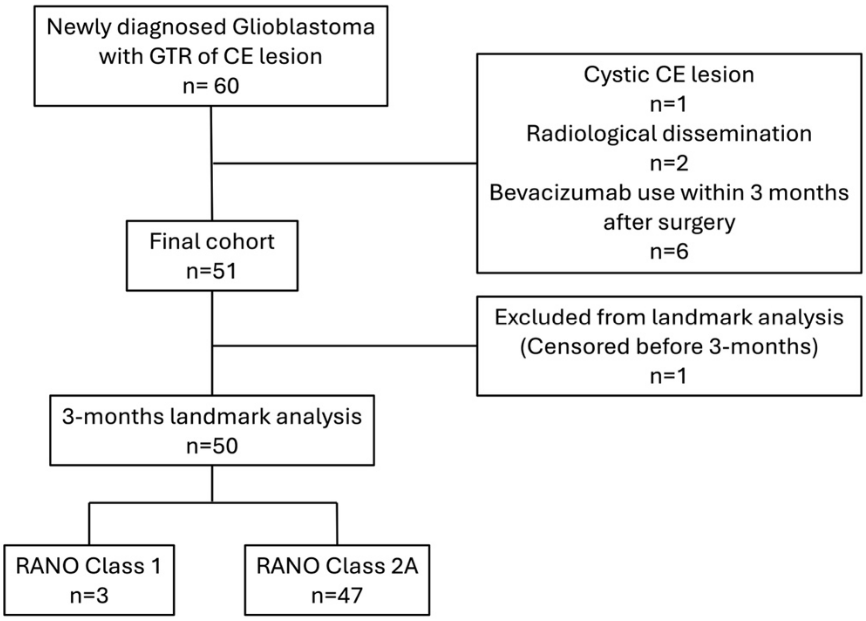

Figure S2. Patient enrollment and exclusion flowchart. Of 60 consecutive patients with newly diagnosed glioblastoma who underwent gross total resection of the contrast-enhancing lesion, 9 were excluded according to prespecified criteria, leaving 51 patients in the final cohort. One patient was further excluded from the 3-month landmark analysis due to censoring before 3 months, resulting in 50 patients for the primary analysis. Among these, 3 were classified as RANO Class 1 and 47 as RANO Class 2A.

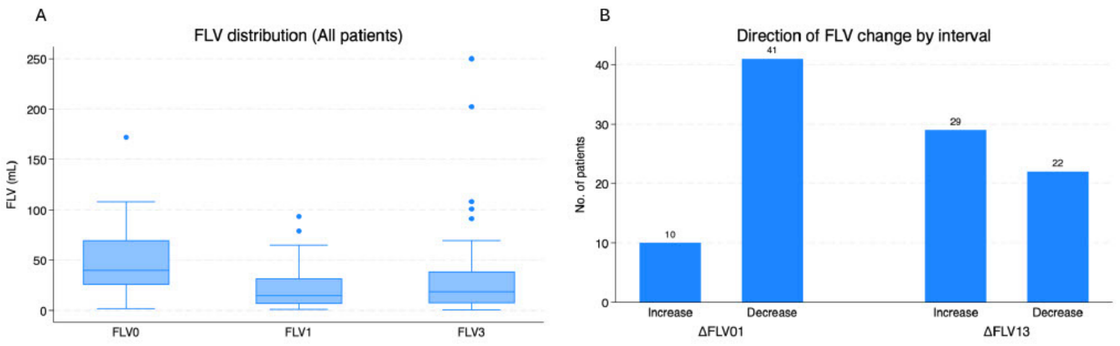

Figure S3. Distribution and direction of postoperative FLV changes. (A) Box-and-whisker plots of FLV0, FLV1, and FLV3 in the full cohort. The boxes indicate the interquartile range with the median line; the whiskers represent 1.5×IQR; and the points denote outliers. (B) The direction of FLV change by interval. In the early interval (ΔFLV0-1), most patients showed a decrease (41/51) and a minority showed an increase (10/51). In the late interval (ΔFLV1-3), decreases and increases coexisted (22/51 and 29/51, respectively), which indicated heterogeneous trajectories across patients.

Table S1: Baseline volumetric MRI measures

	Median volume (mL)	IQR
CEV PreOp	32.6	12.4-58.1
FLV PreOp	53.5	31.7-79.1
FLV0	40.2	25.3-69.7
FLV1	15.0	6.52-32.0
FLV3	18.9	7.15-39.0

Table S2. Univariable Cox regression at the 3-month landmark (overall survival)

Variable	HR	95% CI	p-value
Δ FLV0-1[10-pp]	0.89	0.821-0.973	0.010

ΔFLV1-3[10-pp]	0.99	0.985-0.999	0.024
Age >65 (yes vs no)	2.28	1.173-4.419	0.015
Sex (male vs female)	1.36	0.724-2.544	0.336
p*MGMT* hypermethylation (yes vs no)	0.25	0.125-0.484	<0.001
Post-op KPS ≥80 (yes vs no)	1.10	0.527-2.303	0.800
RANO risk score (low vs intermediate)	0.36	0.171-0.749	0.004

HR reflect a 10-pp absolute reduction in FLV.

Table S3. Overall survival at the 3-month landmark: dichotomized FLV reduction (≥20% vs <20%).

Variable	HR	95%CI	p-value
Early ≥20% vs <20%	0.33	0.14-0.76	0.010
Late ≥20% vs <20%	0.48	0.20-1.13	0.093

In the multivariable Cox model including both early and late FLV reduction (≥20%), early reduction within 1 month after surgery was independently associated with improved overall survival, whereas late reduction between 1–3 months showed only a marginal association.

Table S4. Multivariable Cox model at the 3-month landmark (OS) in RANO Class2 (47 patients with 39 death events/ 39 deaths).

Variable	HR	95% CI	p-value
ΔFLV0-1[10-pp]	0.90	0.82-0.99	0.037
FLV0 ≤ 40ml (yes vs no)	0.75	0.37-1.55	0.438
Age ≤65y (yes vs no)	0.36	0.13-0.73	0.005
p*MGMT* hyper methylation (yes vs no)	0.26	0.13-0.52	<0.001
Postope KPS ≥ 80 (yes vs no)	1.09	0.41-2.89	0.864

HRs were estimated using a Cox proportional hazards model in the RANO resect classification Class 2 subgroup with a 3-month landmark (time-at-risk starting at 3 months after surgery). ΔFLV0-1 was modeled as a continuous predictor per 10- pp greater reduction. The model adjusted for postoperative residual FLV0 dichotomized at 40 mL (≤40 vs >40 mL), age (≤65 years), p*MGMT* methylation status, and postoperative KPS (≥80).

Table S5. Association between p*MGMT* methylation status and ΔFLV01 in RANO-resect class2.

	Hyper methylation (n=17)	Hypo methylation (n=34)	
early ≥20%	14	20	p=0.168
early <20%	2	11	

Cross-tabulation of p*MGMT* methylation status and early FLV reduction during the early postoperative interval (0–1 month), dichotomized at a 20% reduction threshold (≥20% vs <20%). Patients with ΔFLV01 ≥20% showed a numerically higher proportion of p*MGMT* methylation compared with those with ΔFLV01 <20%; however, this difference was not statistically significant (Fisher's exact test, $p = 0.168$).

Abbreviations

CE	Contrast-enhancing
CEV	Contrast-enhancing tumor volume
CI	Confidence interval
FLV	FLAIR lesion volume
FLV0	Immediate postoperative FLV
FLV1	FLV at 1 month
FLV3	FLV at 3 months
FLAIR	Fluid attenuated inversion recovery
HR	Hazard ratio
IQR	Interquartile range
KPS	Karnofsky performance status
MGMT	O6-methylguanine-DNA methyltransferase
MRI	Magnetic resonance imaging
OS	Overall survival
PFS	Progression free survival
pp	Percentage points
PreOp	Preoperative
RANO	Response Assessment in Neuro-Oncology
RT	Radiotherapy
TMZ	Temozolomide
